# Membrane Technologies for Bioengineering Microalgae: Sustainable Applications in Biomass Production, Carbon Capture, and Industrial Wastewater Valorization

**DOI:** 10.3390/membranes15070205

**Published:** 2025-07-11

**Authors:** Michele Greque Morais, Gabriel Martins Rosa, Luiza Moraes, Larissa Chivanski Lopes, Jorge Alberto Vieira Costa

**Affiliations:** 1 Laboratory of Microbiology and Biochemistry, College of Chemistry and Food Engineering, Federal University of Rio Grande, Rio Grande 96203-900, RS, Brazil; 2 Laboratory of Biochemical Engineering, College of Chemistry and Food Engineering, Federal University of Rio Grande, Rio Grande 96203-900, RS, Brazil

**Keywords:** microalgae biorefinery, membrane photobioreactors, porous membrane materials, sustainable development, environmental technologies

## Abstract

In accordance with growing environmental pressures and the demand for sustainable industrial practices, membrane technologies have emerged as key enablers for increasing efficiency, reducing emissions, and supporting circular processes across multiple sectors. This review focuses on the integration among microalgae-based systems, offering innovative and sustainable solutions for biomass production, carbon capture, and industrial wastewater treatment. In cultivation, membrane photobioreactors (MPBRs) have demonstrated biomass productivity up to nine times greater than that of conventional systems and significant reductions in water (above 75%) and energy (approximately 0.75 kWh/m^3^) footprints. For carbon capture, hollow fiber membranes and hybrid configurations increase CO_2_ transfer rates by up to 300%, achieving utilization efficiencies above 85%. Coupling membrane systems with industrial effluents has enabled nutrient removal efficiencies of up to 97% for nitrogen and 93% for phosphorus, contributing to environmental remediation and resource recovery. This review also highlights recent innovations, such as self-forming dynamic membranes, magnetically induced vibration systems, antifouling surface modifications, and advanced control strategies that optimize process performance and energy use. These advancements position membrane-based microalgae systems as promising platforms for carbon-neutral biorefineries and sustainable industrial operations, particularly in the oil and gas, mining, and environmental technology sectors, which are aligned with global climate goals and the UN Sustainable Development Goals (SDGs).

## 1. Introduction

Microalgae constitute a promising biotechnological alternative with the potential to transform multiple industrial sectors. These organisms have the capacity to generate value-added products and mitigate environmental impacts [1]. These photosynthetic microorganisms are distinguished by their accelerated growth rates, efficiency in CO_2_ fixation, ability to valorize wastewater, and ability to produce biomass rich in compounds of commercial interest, including lipids, proteins, carbohydrates, and bioactive secondary metabolites [2,3]. The flexibility of microalgae permits their application in a variety of sectors, including biofuels, human nutrition, wastewater treatment, and carbon sequestration [4,5]. Moreover, microalgae have been shown to sequester approximately twice the amount of CO_2_ per unit of dry biomass produced, highlighting their potential to mitigate industrial emissions [6].

Despite their potential, the commercial implementation of microalgae-based developments faces challenges such as limitations in energy efficiency, biomass volumetric productivity, economical cultivation methods and downstream processing [7]. In this context, the employment of membrane technologies has emerged as a strategy to address bottlenecks experienced throughout the microalgae value chain. These technologies are based on selective barriers that allow the preferential passage of certain components while retaining others, offering significant advantages in terms of biomass separation, concentration and purification. In this context, significant progress has been made in the fields of membrane materials, configurations, and processes, leading to substantial expansion of their application potential in the domain of microalgae [8].

Membrane technologies cover several functions in the microalgae biotechnology value chain. In the context of cultivation, membrane photobioreactors (MPBRs) have been shown to facilitate the efficient retention of biomass while enabling recirculation of the medium. This optimizes the use of nutrients and water, thereby increasing the overall efficiency of the process. In harvesting, membrane filtration processes offer energy-efficient alternatives to centrifugation and other conventional techniques. Membranes can improve the economics of downstream processing by facilitating the extraction, fractionation, and purification of compounds of interest [8,9].

The application of membrane technologies in microalgae systems has gained prominence in sectors such as oil and gas, mining and environmental technologies, especially in carbon capture, effluent treatment and resource recovery [8,10]. These solutions contribute to the reduction in greenhouse gas (GHG) emissions, the sustainable use of water and the promotion of a circular bioeconomy, in line with global sustainability goals related to access to water, energy transition, industrial innovation, responsible production patterns and climate change mitigation.

While membrane technologies have been previously reviewed, many existing studies tend to focus on isolated applications or fail to provide an integrated perspective that combines biomass production, carbon capture, and circular resource recovery within the bioengineering field of microalgae. Furthermore, recent technological innovations—such as self-forming dynamic membranes, electro-Fenton systems, and advanced control strategies—have not yet been comprehensively analyzed within a unified framework. Therefore, this review aims to bridge these gaps by offering a comprehensive overview of the state-of-the-art in membrane-based microalgae systems, with an emphasis on sustainability and their integration into industrial processes.

## 2. Membrane Technologies for Microalgae-Based Systems

**Figure 1:**
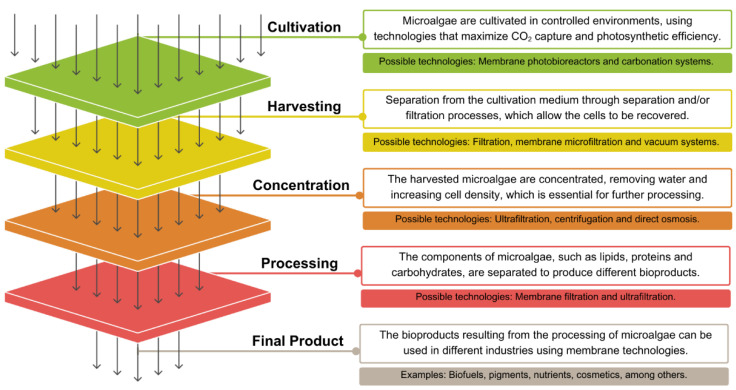
Use of membrane technology in the production chain of microalgae and bioproducts.

Membrane-based systems have been identified as a solution to the main limitations of conventional microalgae cultivation processes, offering improvements in biomass productivity, water and nutrient utilization efficiency, and process stability. The integration of selective barriers that facilitate the regulated separation of biomass from the culture medium is a key feature of membrane technology. This enables the maintenance of high biomass concentrations, the enhancement of photosynthetic performance, and the optimization of resource recovery (Figure 1).

Membrane photobioreactors (MPBRs) have been extensively investigated for their ability to enhance microalgae cultivation by combining biomass retention and carbon transfer functions. In addition, dedicated biomass retention mechanisms based on membrane filtration contribute significantly to the operational performance of cultivation systems, minimizing washout, providing relatively high biomass concentrations and supporting continuous or semicontinuous process configurations.

### 2.1. Membrane Photobioreactors (MPBRs)

The integration of membrane separation technologies with conventional photobioreactor principles in membrane photobioreactors (MPBRs) suggests substantial advancements in microalgae cultivation systems. The primary function of the membrane is to distinguish between the two main configurations of MPBRs [11]. In the photobioreactor configuration with membrane biomass retention (BR-MPBR), the membrane functions as a selective barrier, selectively retaining microalgal cells while allowing the cultivation medium to pass through. This configuration is critical for maintaining high cell concentrations and efficient nutrient utilization [12]. Conversely, in membrane carbonation photobioreactors (C-MPBRs), the transfer of CO_2_ for microalgae cultivation can occur through membrane contactors or diffusers [13,14]. Research has demonstrated that the implementation of membranes in the mass transfer of CO_2_ for microalgae cultivation enhances the efficiency of gas utilization and biomass productivity and minimizes losses to the atmosphere [15,16].

Microalgal cultivation methods that utilize MPBR technology have demonstrated clear advantages over more conventional methods. Quantitative comparative analyses demonstrate significant enhancements in various performance metrics, including biomass productivity, which has been shown to reach up to nine times the level observed in conventional systems [17]. The efficient retention of biomass by membranes has been demonstrated to increase the typical concentrations of the parameter in conventional open systems by a factor of two [18]. Moreover, in comparison with conventional systems, MPBRs reduce water consumption by up to 77%, which is a result of the efficient recirculation of the medium following membrane separation [17]. This characteristic is particularly important in regions characterized by water scarcity or when costly growing media are utilized [13]. Consequently, the increased cell concentration leads to increased volumetric productivity and more efficient utilization of space (Table 1).

The performance of MPBRs is influenced by the optimization of interrelated operating parameters, among which the light path, retention time, permeate flow, and other parameters stand out. The balance between high cell concentration and light availability represents a challenge in MPBRs. The formation of dark zones, regions with insufficient exposure to photosynthetically active radiation (PAR), has been observed under high-cell-concentration conditions. This phenomenon can compromise the efficiency of the system.

Photobioreactors with a reduced light path (10 cm versus 25 cm) provide an increase of approximately 200% in biomass productivity and 70% in photosynthetic efficiency because of the more homogeneous penetration of light. This optimization of light distribution minimizes dark zones and photoinhibition, resulting in a more efficient use of light energy by microalgal cells [22]. Mitigation strategies for this phenomenon include the design of reactors with a reduced optical path (~10 cm) and the implementation of dynamic adjustments to the solids retention time (SRT) in response to variations in the environmental light intensity [21,22].

In MPBRs, the control of design parameters, particularly hydraulic retention time (HRT) and SRT (also referred to as biomass retention time, BRT), is essential to ensure optimal system performance. The ability to decouple these retention times is one of the main advantages of MPBRs over conventional photobioreactors, allowing the HRT to be adjusted to increase the treatment capacity and loading rate, whereas the SRT is maintained to optimize the biomass concentration and quality. However, this operational flexibility also introduces challenges related to balancing the maximization of biomass production with nutrient removal efficiency [23].

Adjusting operating parameters such as the HRT and SRT can control the generation rates of extracellular and intracellular organic matter, influencing fouling characteristics and mechanisms. Membrane fouling control systems that apply localized shear rates or cleaning can minimize cell breakdown and reduce the overall strength of the gel and cake layers [24]. The optimal SRT typically ranges from 3.0 to 4.5 d, and the ideal HRT is between 1.3 and 1.5 d, which optimizes cell growth, prevents biomass decomposition, and maintains adequate nutrient loading in membrane-based microalgae cultivation systems treating synthetic municipal wastewater. This interval has been shown to enhance both nutrient removal and biomass concentration. The ideal SRT may vary depending on the specific microalgal strain and the characteristics of the wastewater. On the other hand, in wastewater treatment applications, moderate SRT values (ranging from 15 to 20 d) are recommended to promote stable growth, whereas SRTs exceeding 25 d may lead to excessive self-shading and reduced photosynthetic efficiency [25].

Studies have shown that the increase in productivity resulting from a reduced HRT can compromise nutrient removal efficiency, highlighting a trade-off between biological performance and treatment efficiency [18,26]. Significant deviations from these values can result in system instability, excessive biomass accumulation, or nutrient washout [27]. The high cell concentration combined with prolonged HRT results in a superior nutrient utilization efficiency in MPBR systems, with high nitrogen (7.7 mg L^−1^·d^−1^) and phosphorus (1.2 mg L^−1^·d^−1^) removal rates [21]. Membrane-based bioreactors demonstrate efficiency in the simultaneous removal of these nutrients from industrial wastewater, often achieving removal efficiencies above 95% for ammonium nitrogen and total phosphorus [9,17].

Maintaining moderate permeate fluxes (15 to 26 L m^−2^ h^−1^) combined with regular backwashing cycles (40 s every 10 cycles) significantly reduces membrane fouling without compromising productivity. Excessively high fluxes accelerate fouling, and excessively low fluxes underutilize the membrane’s capacity. The specific gas demand (between 16 and 20 Nm^3^ m^−3^) represents an optimal balance for efficient membrane cleaning and sustainable energy consumption. A lower aeration results in accelerated fouling accumulation, whereas higher values increase energy consumption without proportional benefits [22].

Compared with conventional systems, MPBR systems can reduce mechanical aeration requirements by up to 60%, and when operating under optimized conditions, they ensure stable long-term productivity and enhanced energy efficiency. Magnetically induced vibration systems can be used in this photobioreactor configuration. These systems use magnetic fields to induce controlled vibration in the membranes. The vibration in these systems creates shear stresses that destabilize the boundary layer and reduce fouling, enabling continuous operation at high fluxes. Magnetically induced membrane vibration (MMV) systems operate with significantly lower energy consumption (~0.77–0.84 kWh m^−3^) than conventional configurations do while ensuring effective fouling control and superior economic viability [19].

Advances in materials science have expanded the possibilities for optimizing membrane bioreactors (MPBRs). Adding metallic nanoparticles to the membrane material can reduce fouling by up to 40%, thereby increasing the system’s operational lifetime [28]. The development of membranes with hydrophilic/hydrophobic gradients improves selectivity and reduces unwanted biofilm formation [9].

An emerging configuration within the MPBR family is the algae-based membrane bioreactor (AMBR), which integrates microalgal cultivation with membrane-based wastewater treatment. The AMBR has demonstrated promising results in coupling nutrient removal and biomass production under real effluent conditions. This hybrid system promotes circular resource recovery and enhances effluent polishing while maintaining operational stability. The integration of microalgae with membrane separation units in AMBR offers the advantages of improving water quality and generating valuable biomass for downstream applications [10].

### 2.2. Biomass Retention Mechanisms

Membrane systems for biomass retention (BR-MPBRs) evolved from conventional MPBRs with a focus on optimizing the separation and retention of microalgal biomass. In these systems, the membranes act as selective barriers, providing precise control over microalgae in the bioreactor regardless of the dilution rate of the cultivation medium [8]. The separation mechanisms in BR-MPBRs are based on the fundamental principles discussed below.

Size exclusion, which is carried out mainly by microfiltration and ultrafiltration membranes, selectively retains cells on the basis of their physical dimensions. This process allows only components of the medium with a molecular size below the cutoff limit to permeate the membrane. This mechanism effectively separates suspended solids and larger colloids [29]. For most microalgal species, membranes with pore sizes between 0.1 and 1.0 μm efficiently retain cells without excessively restricting flow [30,31].

Physicochemical interactions, such as electrostatic and hydrophobic interactions, play a significant role in membrane fouling and biomass retention [29,32]. However, extracellular polymeric substances (EPSs), which are composed mainly of polysaccharides and proteins, are the primary fouling agents due to their hydrophilic nature and gelling properties [29]. The composition of EPSs varies among microalgal species and cultivation conditions. Microalgal species known for high EPS production, such as *Chlorella vulgaris* and *Botryococcus braunii*, often demonstrate accelerated fouling rates, whereas *Spirulina platensis* and *Dunaliella salina* typically demonstrate a lower propensity for this phenomenon [33].

Advances in this field include the development of self-forming dynamic membranes (SFDMs), which use microalgal biomass to form a primary filtration layer on a low-cost, macroporous support. These systems significantly reduce membrane investment and often demonstrate better flow recovery after cleaning because of the regenerative nature of the filter layer. This approach is a promising solution for regions where membrane technology poses a significant economic barrier [34].

The implementation of membrane biomass retention systems (BR-MPBRs) can considerably impact the productivity and quality of microalgal biomass. BR-MPBR systems can maintain higher biomass concentrations (~5 g L^−1^) than can conventional systems [21]. This improves the downstream process by retaining a relatively high biomass concentration and enabling greater volumetric productivity [35,36]. These systems exert selective control over bacterial populations and cell viability, which are influenced by operating conditions such as the solids concentration and retention time [37].

### 2.3. Biofilm-Based Systems

Adhered cultivation systems use membranes or modified surfaces to support the growth of microalgal biofilms. In this configuration, cells adhere to and grow on the designed surfaces, forming dense biofilms that optimize light capture and nutrient transfer [20]. Porous membranes are essential for compartmentalized cell cocultures and barrier support, mimicking cell–cell interactions at the nanometer scale. However, available membranes have limitations, such as a high thickness, low porosity, and random pore distribution, which can distort physiological multicellular interactions [38].

According to Zhang et al. [39], the capacity of membranes to support cell adhesion must be evaluated to enable the use of biofilm-based photobioreactors in wastewater treatment. In this study, the authors evaluated five types of microfiltration membranes (polypropylene, polyamide, fiberglass, cellulose nitrate, and cellulose acetate) as support materials for the adherent cultivation of *Chlorella pyrenoidosa*. The results showed that *C*. *pyrenoidosa* had a more hydrophobic surface and tended to agglomerate, forming a dense biomass layer. The interactions between *C*. *pyrenoidosa* and the cellulose acetate and cellulose nitrate membranes were more intense than those with the other membranes were, providing greater stability in terms of cell adhesion and biomass accumulation (~60 and 51 g m^−2^, respectively).

The adhered cultivation system with artificial vertical supports enables the arrangement of multiple microalgae films in a matrix configuration, thereby maximizing the surface area-to-volume ratio of the reactor [20]. This three-dimensional arrangement optimizes light capture, nutrient access, and gas exchange simultaneously. Materials incorporating graphene-based membranes are highly stable both mechanically and chemically, promoting greater cell adhesion and nutrient transfer efficiency [8]. These nanostructured surfaces provide optimized microenvironments for the formation of high-density biofilms.

Cultures that adhere to membranes have the highest biomass productivity (50–80 g m^−2^ d^−1^), which exceeds that of conventional suspended systems (10–30 g m^−2^ d^−1^). The photosynthetic efficiency of microalgae (5.2–8.3%) approaches the theoretical limit for photosynthetic organisms (~10%), far exceeding what is common in open systems (1.5–3.0%). The biofilm cell density can be up to ten times greater than that in suspension cultures, optimizing space and infrastructure use [20].

Adhered cultivation systems have specific advantages over conventional systems for certain applications. The production of biofuels is possible due to high biomass productivity and concentration, as productivity per area is a critical economic factor. Additionally, inducing controlled stress in biofilms can promote lipid accumulation in oleaginous microalgal species [40]. Direct exposure to air facilitates the efficient capture of atmospheric CO_2_ or industrial gaseous effluents, making these systems suitable for airborne effluent bioremediation applications [40].

Meng et al. [41] evaluated the impact of membrane blockage on biomass productivity and lipid accumulation in the cultivation of adhered *C. vulgaris* cells in a vertical system with cellulose acetate membranes varying in porosity (0.45, 1.2, 2.0 and 3.0 µm). The highest biomass productivity (5.02 g m^−2^ d^−1^) was achieved with a pore diameter of 1.2 µm; however, this parameter decreased with increasing porosity due to complete blockage by cells. The lipid content was also influenced by pore size due to variations in nutrient transfer resistance and light intensity. The highest lipid accumulation (23.7% *w*/*w*) occurred with the 1.2 µm membrane and was associated with stress due to nitrogen limitation and efficient illumination of the cultures. Cell adhesion was unaffected by the surface properties of the membrane because inoculation was carried out by filtration. Biomass productivity remained consistent during recultivation, demonstrating the potential for reuse of the membranes.

## 3. Carbon Capture via Membrane-Based Systems

Biological carbon capture via microalgae represents a promising strategy for mitigating GHG emissions by combining CO_2_ biofixation with biomass production. Membrane-based technologies have improved this approach by overcoming the critical limitation of low gas–liquid mass transfer efficiency in conventional systems. The membrane systems operate through distinct mechanisms that maximize CO_2_ availability to microalgae cells while minimizing losses and increasing photosynthetic efficiency [16,42,43,44].

### 3.1. Direct CO_2_ Transfer via Membranes in Microalgae Cultures

**Figure 2:**
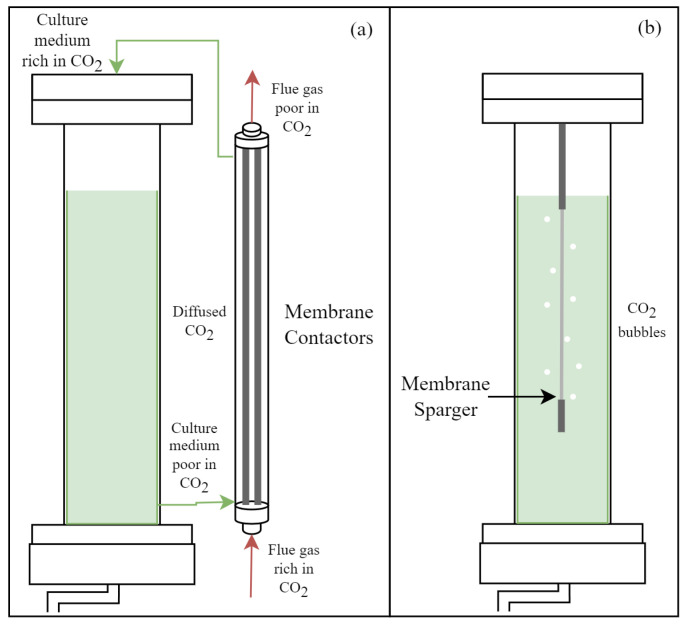
Membrane carbonation photobioreactors (C-MPBR): (**a**) membrane contactor; (**b**) membrane diffuser (sparger).

Membrane photobioreactors for carbonation (C-MPBRs) have become notable for their high CO_2_ transfer efficiency, which is often greater than 80%. This contrasts with the 30–40% efficiency typical of conventional bubbling systems. This efficiency allows for greater utilization of dissolved inorganic carbon, which is a critical factor for the economic and environmental sustainability of the process [8]. These systems can supply CO_2_ for microalgae cultivation via two approaches: (i) membrane contactors, where the membrane acts as an interface between the gas and liquid phases to promote dissolved CO_2_ diffusion via a concentration gradient without bubble formation, or (ii) membrane diffusers (spargers), which generate uniform microbubbles to increase the interfacial area and CO_2_ dissolution efficiency [13,14] (Figure 2).

Nonporous (dense), hydrophobic, hollow or flat fiber membranes composed of polydimethylsiloxane (PDMS) or polyurethane are widely used in membrane contactors to promote the controlled and efficient transfer of CO_2_ to microalgal cultures. In these systems, CO_2_ is transferred exclusively by molecular diffusion without bubble formation, maximizing the contact time and increasing the dissolution efficiency [45]. This configuration also results in low gas losses to the atmosphere and minimizes hydrodynamic stress on microalgal cells [13]. Kim et al. [16] evaluated a C-MPBR composed of dense polyurethane hollow fiber membranes to supply CO_2_ by diffusion to *Synechocystis* sp. PCC6803 cultures. The authors reported that increasing the liquid recirculation flow rate increased the biomass productivity of microalgae by 26.2% and the dissolved inorganic carbon (DIC) concentration by 237.9%. The CO_2_ utilization efficiency reached 88%, with minimal gaseous losses to the atmosphere.

Zheng et al. [46] used a composite membrane consisting of a porous polysulfone support layer coated with a thin, nonporous polydimethylsiloxane (PDMS) layer. This configuration formed a liquid–liquid contactor capable of transferring CO_2_ from solvents loaded with the gas (potassium carbonate, monoethanolamine, and potassium glycinate). The hydrophobic, nonporous nature of the membrane’s active layer allowed efficient CO_2_ diffusion without bubble formation in both freshwater and saltwater culture media. The saltwater medium supported more efficient CO_2_ delivery, which was attributed to its higher buffering capacity, 150% higher DIC concentration, and reduced solvent dilution from lower water permeation.

Xu et al. [43] investigated CO_2_ transfer in *Nannochloropsis* sp. cultures via flat membranes composed of polydimethylsiloxane (PDMS) with a polysulfone support operating in a liquid–liquid contactor. With pH control, they reported values of 90% CO_2_ utilization efficiency and 0.10 g L^−1^ d^−1^ biomass productivity, which were higher than those obtained with air bubbling (0.03 g L^−1^ d^−1^ and 10.7% efficiency). Reducing the operating time of the solvent pump by up to 97% reinforces the potential of these membranes as an efficient, energy-saving solution for controlling the supply of carbon in photobioreactors.

Porous hollow fiber membranes, typically made from hydrophobic polymers such as polytetrafluoroethylene (PTFE), polyvinylidene fluoride (PVDF), and polyethersulfone (PES), are commonly used as spargers for supplying CO_2_ to microalgal cultures. In these systems, the gas disperses through the micrometer-sized pores of the membrane to form fine, uniform microbubbles. This significantly increases the interfacial contact area compared with that of conventional bubbling systems, which generate larger bubbles with lower mass transfer efficiency [13,14].

Although membrane diffusers promote less intense mixing, they are more efficient at transferring CO_2_ due to their enlarged contact surface. Fan et al. [14] used microporous polyethersulfone (PES) hollow fiber membranes as spargers to supply carbon dioxide for *C. vulgaris* cultivation. This system generated uniform microbubbles, which significantly increased the gas–liquid contact area. Despite the lower mixing intensity compared with conventional bubbling, the membrane sparger configuration demonstrated higher CO_2_ fixation efficiency, achieving a biofixation rate of up to 3.6 g L^−1^ d^−1^ and a mass transfer coefficient (k_L_a) of approximately 0.13 s^−1^. These results highlight the potential of membrane spargers to optimize carbon transfer while reducing the hydrodynamic impact and favoring strains that are sensitive to turbulence.

This system achieved a fixation rate of 0.23 g L^−1^ d^−1^ and an efficiency of 80.5% when CO_2_ was used, which significantly exceeded the performance of conventional spargers. Additionally, better pH stability, greater CID accumulation, and reduced chemical gradients in the cultivation medium were observed.

Another example of the use of membrane spargers was presented by Moraes et al. [47], who developed a system incorporating hollow polyetherimide fibers into a tubular photobioreactor to deliver CO_2_ to *Spirulina* sp. LEB 18 cultures. The system achieved a CO_2_ fixation rate of 0.23 g L^−1^ d^−1^ and an efficiency of 80.5%, substantially outperforming conventional spargers. In addition, it provided improved pH stability (maintaining values below pH 10) and a 33.5% greater accumulation of DIC, along with a dominant bicarbonate fraction reaching 98.9%, increasing carbon availability and uptake.

### 3.2. Selective Separation of CO_2_ from Flue Gases

The selective separation of CO_2_ in complex gas mixtures via several types of membranes, including dense polymers, facilitated transport, and mixed membranes, has been widely explored [48,49]. Selectivity is important for applications involving industrial gaseous effluents, which usually contain 10–15% CO_2_ mixed with potentially toxic compounds for microalgae. Compared with conventional pretreatment approaches, the use of selective membranes can reduce the total cost of capturing this greenhouse gas (GHG) in industrial applications by up to 40%. This advantage stems from the simplification of processes and the significant reduction in auxiliary equipment investment for gas treatment [50,51].

Dense polymer membranes operate via a solution–diffusion mechanism. In this mechanism, gases dissolve in the polymer matrix and diffuse selectively according to their physicochemical properties. CO_2_ selectivity results from its greater affinity and mobility in the matrix than other mixture components, such as N_2_ or CH_4_ [48]. Kerner et al. [44] demonstrated the application of a PolyActive™ copolymer membrane to concentrate CO_2_ from flue gases originating from the burning of natural gas (97% CH_4_), which contained approximately 9% CO_2_. The membrane increased the CO_2_ concentration in the permeate to 48%, with a recovery efficiency of 62%. The enriched gas was used to cultivate *Chlorella sorokiniana* under stable conditions (pH ~6.2, temperature <28 °C), reducing CO_2_ loss to 24%. The system achieved a productivity rate of 16.5 g m^−2^ d^−1^ and a photosynthetic efficiency rate of 0.54 g mol^−1^ PAR, demonstrating the potential of selective membrane separation to integrate industrial emissions with sustainable microalgal cultivation.

Mixed matrix membranes (MMMs) consist of polymeric matrices that incorporate dispersed inorganic materials, such as zeolites, metal–organic frameworks, and supported ionic liquids, to increase selectivity, permeability, and fouling resistance. MMMs represent a promising technology for integration into photobioreactors, particularly to improve CO_2_ transfer for microalgal cultivation and biomass production. These additives can improve the selectivity or permeability of the membranes without the need for a direct chemical reaction. Facilitated transport membranes incorporate transport agents such as amines or enzymes that react reversibly with CO_2_, forming transient compounds that promote preferential migration through the membrane [48].

Functionalized membranes are a step forward for applications involving complex gas mixtures, such as industrial combustion gases, because they have an increased affinity for CO_2_. This allows them to selectively permeate other gases that can inhibit the growth of microalgae, such as SO_2_ and NO_x_ [34,52]. However, certain strains, such as *Spirulina* sp. LEB 18, *Scenedesmus obliquus* LEB 22 [53] and *Chlorella fusca* LEB 111 [54], can assimilate SO_2_ and NO_x_ as nutrients, expanding the possibilities for using these effluents in microalgal cultures.

For example, Wang et al. [42] developed a membrane sparger functionalized with carbonic anhydrase (CA) and applied it to an electrospun polysulfone membrane. The enzyme was immobilized by electrostatic adsorption in layers and acted at the gas–liquid interface, where it catalyzed the conversion of CO_2_ into bicarbonate, thereby increasing its solubility in the medium. In a raceway reactor, this system increased CO_2_ capture rates by 106% compared with an open tube and by 70% compared with a porous diffuser. There was also a 22% increase in the microalgal biomass concentration. The stability of the enzymatic activity for over 30 d further supports the viability of this system as a low-energy solution for carbon biofixation.

### 3.3. Selective Bicarbonate Supply and Ionic Regulation with Electrolysis Membranes

Ion electrolysis membranes (IEMs) enable the selective control of the ionic composition of a culture medium through the targeted separation of anions, such as bicarbonate (HCO_3−_), and the exclusion of undesirable cations, such as sodium (Na^+^), within electrochemical systems. This process favors pH regulation and the supply of DIC for microalgal growth. Hou et al. [55] demonstrated this approach by developing an innovative three-chamber system that integrates electrolysis and IEMs to selectively supply bicarbonate to the *C. vulgaris* cultivation medium. In this system, HCO_3−_ ions generated in the cathode chamber were transferred to the central chamber via an anion exchange membrane, and H^+^ ions from the anode stabilized the pH. This configuration enabled operation at a high DIC concentration while avoiding the accumulation of Na^+^ and mitigating ionic stress. Additionally, the cell concentration increased by 403% compared with that of the control system.

In another example of MEI application, Kim et al. [56] developed a two-chamber electrolytic cell equipped with an anion exchange membrane for cultivating *Chlorella* sp. The system used a cathode solution containing sodium bicarbonate (NaHCO_3_), allowing HCO_3−_ ions to migrate selectively into the cultivation medium while retaining Na^+^ cations. Concurrently, H^+^ ions generated in the anode chamber migrated into the bioreactor, promoting continuous pH regulation of the microalgal culture. This configuration eliminates the need to add gaseous CO_2_ or perform chemical corrections with acids, maintaining a stable pH over time. The biomass concentration of *Chlorella* sp. was three times greater than that obtained in conventional systems with pH control. This finding demonstrates the potential of electrochemical technology associated with membranes to maximize microalgal growth with minimal operational intervention.

## 4. Integration of Membrane-Based Systems for Wastewater Treatment and Circular Resource Recovery

Integrating membrane-based technologies with microalgal systems is an innovative, sustainable approach that combines efficient wastewater treatment, carbon capture, and biomass valorization. These hybrid systems promote the removal of pollutants and GHGs by combining selective separation processes with biological conversion routes. They also enable the generation of biomass with potential applications in bioenergy and high-value bioproducts. This section discusses the main configurations that exemplify this integration and highlights their strategic role in promoting circular economies and mitigating environmental impacts in industrial and urban contexts.

### 4.1. Membrane Photobioreactors in Wastewater Treatment: Nutrient Recovery and Biomass Production

The integration of MPBR with microalgae cultivation has proven to be a promising strategy for wastewater treatment across various sectors, simultaneously enabling the removal of nutrients—particularly nitrogen and phosphorus—and microalgal biomass production [18,23,35,36,57,58]. Furthermore, the use of microalgae–bacteria consortia in MPBRs has been investigated for enhanced wastewater treatment [59].

Marbelia et al. [18] reported that the complete retention of *C. vulgaris* biomass within the MPBR, combined with lower HRT values (higher dilution rates: D = 0.5 d^−1^), resulted in a 3.5-fold increase in biomass concentration and a twofold increase in productivity compared with those of a conventional photobioreactor. However, the decoupling of the HRT and SRT revealed a trade-off: the increase in biomass productivity led to a reduction in nutrient removal efficiency.

Yang et al. [26] evaluated the effect of HRT on the operation of an MPBR (with a PVDF microfiltration membrane) cultivating *Chlorella protinosa* with secondary effluent. Reducing the HRT from 24 to 8 h resulted in a 230% increase in biomass productivity, with the ammonia removal efficiency reaching approximately 99%. On the other hand, the reduction in HRT led to a decrease in total nitrogen (TN) removal from approximately 74% to 66% and in chemical oxygen demand (COD) removal from 83% to 70%.

Nguyen et al. [35] investigated the effects of different SRTs (5, 10, and 15 d) on the performance of an MPBR operated with *C. vulgaris* cultivated in synthetic municipal wastewater. Increasing the SRT from 5 to 15 d led to an increase in the biomass concentration from 0.45 to 0.87 g L^−1^, in addition to increasing the nutrient removal efficiency. At an SRT of 15 d, the removal rates reached 86.3% for total nitrogen (TN) and 95.7% for total phosphorus (TP). This study demonstrated that moderate SRTs can simultaneously optimize nutrient recovery and biomass production in MPBRs.

An MPBR system equipped with flat-sheet PVDF membranes was used to cultivate *C. vulgaris*, which was operated with an SRT of 50 d for the treatment of synthetic municipal wastewater. Until 22 days of cultivation, the system exhibited efficient nutrient removal (76.7% for TN and 66.2% for TP). However, from 23 days onward, a reduction in the biomass concentration (from 3.48 to 1.94 g L^−1^) and chlorophyll-a content (from 34.6 to 10.7 mg g^−1^) was observed. Biomass accumulation leads to self-shading and intraspecific competition, compromising effluent quality [58].

As highlighted, although shorter HRTs increase the influent processing rate and may increase microalgal biomass productivity, they often compromise nutrient removal efficiency and can intensify membrane fouling due to increased organic loading and increased filtration frequency [18]. On the other hand, prolonged SRTs, while promoting microalgal biomass accumulation, are also associated with increased release of algal organic matter, which further contributes to membrane fouling [57]. Table 2 presents studies that have investigated the relationships between HRT and SRT and the occurrence of fouling, as well as the control strategies adopted.

### 4.2. Integrated Membrane Photobioreactors for Carbon Capture, Wastewater Treatment, and Biomass Valorization

MPBRs represent advanced hybrid systems that simultaneously support microalgal cultivation, nutrient recovery, CO_2_ capture, and biomass valorization. Unlike the preceding sections, which address these applications individually, this section focuses on integrated configurations that combine these functions into cohesive platforms for sustainable bioindustrial use.

When coupled with wastewater treatment processes, MPBRs have demonstrated high efficiency in removing both CO_2_ and nutrients such as nitrogen and phosphorus—often achieving over 80% CO_2_ capture while generating valuable biomass [18,21,31]. For example, Senatore et al. [31] reported an 80% CO_2_ removal rate from a gas stream containing 15% CO_2_ during *C. vulgaris* cultivation. The use of self-forming dynamic membranes (SFDMs) further improved nutrient removal and allowed greater control over pH and dissolved oxygen. These systems are particularly promising for diffuse emission sources (e.g., wastewater treatment plants, landfills, composting units) because they treat effluents while recovering energy and resources from biomass.

Life cycle assessments (LCAs) reinforce this potential: MPBRs have been shown to reduce the carbon footprint of effluent treatment systems by 30–45% while enabling the generation of high-value bioproducts [7]. Zarra et al. [34] reported up to 46% reductions in photooxidant formation and human toxicity impacts when wastewater was used as a nutrient source, along with minor improvements in particulate matter formation (2.5%) and water consumption (3.6%). However, a low biomass productivity was associated with a 10% increase in global warming potential, which sensitivity analyses suggested could be reduced by up to 37% through cultivation optimization. Furthermore, the cumulative energy demand could be lowered by as much as 40%. Complementary evidence highlights that integrated dewatering approaches, such as bioflocculation followed by filtration, can also reduce energy use and CO_2_ emissions during downstream processing [61]. Hollow-fiber MPBR (HFMPB), for example, demonstrated a CO_2_ removal efficiency of up to 85% and biomass production reaching 2.1 g L^−1^ during *S. platensis* cultivation while simultaneously removing 68% of nitrates under low-pressure operation [62].

Integrated systems may also promote indirect CO_2_ biofixation. Through the anaerobic digestion of microalgal biomass, CO_2−_ rich biogas can be captured and reused in microalgal cultivation, establishing a circular carbon route. Compared with conventional anaerobic digestion, this strategy can increase methane yields by 30–45% while minimizing atmospheric CO_2_ losses [7,63].

Takabe et al. [64] illustrated this circular approach by applying polyimide hollow fiber membranes to purify CO_2_ from biogas for subsequent use in native microalgae cultivation. This system delivered ~98% CO_2_ purity without compromising metabolic performance. Zhang et al. [65] further advanced this concept by coupling an MPBR with a PVDF hollow fiber membrane, enabling simultaneous CH_4_ oxidation by methanotrophs and CO_2_ fixation by microalgae via biofilm formation on the membrane. The system increased CO_2_ fixation by 12% and CH_4_ assimilation by 50%, indicating synergetic metabolic interactions in a multipurpose membrane platform.

Overall, the integration of CO_2_ capture, wastewater remediation, and biomass valorization within MPBRs represents a promising strategy for advancing carbon-neutral biorefineries. These platforms optimize resource utilization and energy recovery while promoting environmental compliance and circularity in industrial systems. To provide a clearer comparison of the systems discussed, Table 3 summarizes key parameters, including biomass productivity, CO_2_ removal efficiency, nutrient recovery, and energy demand, across different membrane–microalgae configurations.

## 5. Process Optimization and Technological Innovations

Recent advances in membrane technology have not only improved operational performance but also addressed critical challenges such as fouling, energy consumption, and scalability. This section reviews the technological innovations and optimization strategies that enhance the efficiency, durability, and economic viability of membrane-based systems used for microalgae cultivation and processing.

### 5.1. Innovations in Membrane Materials and Surface Modifications

Advances in membrane materials and surface engineering have been crucial in mitigating fouling, improving selectivity, and extending the lifespan of membranes in microalgae-based systems. Additionally, improvements in membrane configurations have substantially enhanced the performance of microalgae cultivation systems by addressing critical limitations such as fouling, energy consumption, and scalability.

A notable innovation is the development of a novel attached membrane photobioreactor employing modified polyvinylidene difluoride/polyvinyl pyrrolidone (PVDF/PVP) electrospun membranes. The PVDF:PVP membrane with a 2:1 mass ratio at 20% w/w significantly enhanced CO_2_ mass transfer and light–fluid synergy within the reactor. Compared with the commercial PVDF membrane, the modified membrane increased the carbon sequestration rate by 18.6%, reaching 0.33 g L^−1^ d^−1^. Additionally, this optimized membrane exhibited better hydrophilicity, a more uniform fiber structure, and reduced membrane fouling, contributing to a 16.4% higher microalgae growth rate [66].

Membranes with specific surface patterns use PVDF with microtextured surfaces, which significantly reduces their filtration resistance. In these systems, the stable permeance decreased by only 15% (110 L m^−2^ h^−1^·bar^−1^) compared with the 72% observed in conventional flat membranes under identical operating conditions. This improvement is primarily due to the controlled disturbance of the boundary layer and reduced compaction of the cell deposit [67].

The inclined membrane panels are configured at optimized angles relative to the aeration flow rate to promote a better distribution of shear stresses over the membrane surface. This geometry favors fouling control by directing air bubbles more effectively across the active surface. This makes it possible to achieve high average permeances (870 L·m^−2^·h^−1^·bar^−1^) under ideal aeration conditions (1.5 L min^−1^) in the cultivation of *C*. *vulgaris* [68].

The electro-Fenton process, which uses hollow porous carbon membranes containing carbon and iron nanotubes, has demonstrated high efficiency in harvesting *Coelastrum* microalgae. This process mitigates fouling caused by cells and extracellular organic matter. Under an electrical voltage of −1.0 V, the system increased the biomass concentration by 2.5 times and the permeance of pure water by up to 16 times compared with those of the control. After 30 min of regeneration, the permeance fully recovered, increasing the concentration of microalgae from 1.1 g L^−1^ to 7.5 g L^−1^ without damaging the membrane or microalgal cells [69].

These configurations have the potential to overcome the limitations of membrane technologies for microalgae cultivation, particularly those related to fouling and energy consumption.

### 5.2. Strategies for Fouling Mitigation

Membrane fouling is the most persistent and impactful challenge for the sustainable implementation of membrane technologies in microalgae systems and has been identified as a limitation in the literature [33,45,70,71,72]. This phenomenon manifests itself through multiple mechanisms, each of which requires specific mitigation strategies. The primary mechanism of performance reduction in microalgae systems is organic fouling, which is the result of the deposition of EPS, proteins, polysaccharides, and other biomolecules on the membrane surface and pores. This fouling is especially evident in high-cell-concentration cultures, where elevated concentrations of dissolved organic compounds resulting from metabolism and intermittent cell lysis generate complex matrices with high adsorption properties [72].

Advancements in mitigation strategies have led to enhanced membrane performance and operational longevity, resulting in cost reductions and increased commercial viability. Among these methods, pretreatments have proven effective in reducing resistance to filtration. Bioflocculation resulting from natural interactions between microalgae and bacteria, leading to the formation of larger aggregates, has been shown to reduce energy consumption to low values (0.041 kWh kg_biomass_^−1^) [61]. Similarly, the addition of flocculants such as chitosan (5–10 mg L^−1^) modifies the rheological properties of the suspension, facilitating subsequent filtration [67].

Another strategy involves the alteration of surfaces. Membranes with negative surface charges have been demonstrated to reduce the adhesion of microalgae in cultures with a neutral to alkaline pH balance. The implementation of specific microtexturizations has been demonstrated to be an effective strategy for mitigating fouling, resulting in a minimal decrease in permeance of only 15%, in contrast to the 70–80% reduction observed in conventional membranes [8]. Hydrophilic coatings have also been demonstrated to minimize the adhesion of organic matter. The integration of antifouling functionalities directly into the polymer matrix constitutes a promising solution, despite the concomitant increase in production costs [45].

Operational strategies such as filtration–relaxation cycles and periodic backwashing reduce fouling by relieving pressure and removing accumulated biomass [61]. The incorporation of air bubbles into ventilation cycles has been demonstrated to increase surface shear, thereby facilitating the removal of fouling [61], whereas optimizing the tangential flow speed has been shown to maintain filtration efficiency [72]. Advanced cleaning technologies, such as electro-Fenton systems, have been demonstrated to selectively degrade organic compounds, thereby increasing cultivation efficiency by up to 250% [71]. Furthermore, ultrasound-assisted cleaning has been shown to increase biomass removal without the need to employ aggressive chemicals [72].

Together, these complementary strategies allow membrane systems to operate for longer periods of time without significantly compromising flow or increasing transmembrane pressure. This reduces operating costs and increases commercial viability.

### 5.3. Control and Automation Strategies

The development and implementation of advanced control and automation strategies are critical elements in optimizing the performance and sustainability of membrane systems for microalgae cultivation. These control strategies are designed to optimize productivity and efficiency while also enhancing operational robustness in the face of external disturbances. This is a critical feature for commercial implementations, where ensuring production consistency is essential.

Dynamic SRT/HRT adjustments, facilitated by adaptive algorithms, modify retention times in response to environmental variables, particularly light intensity and temperature. For example, a temporary reduction in the SRT (to ~2 d) during periods of low light prevents the formation of dark zones due to excessive cell density, thereby optimizing the use of available radiation [25].

The integration of sensor networks capable of monitoring key parameters—such as optical densities (OD680, OD720), the soluble chemical oxygen demand to volatile suspended solids ratio (sCOD:VSS), nutrient concentrations, and light transmission—enables real-time assessment of microalgal physiological status and reactor performance. In particular, the sCOD:VSS ratio is a reliable indirect indicator of cellular stress and potential culture collapse. This continuous monitoring approach supports the early detection of unfavorable conditions and timely intervention, ensuring the stable operation of MPBRs [73].

Integrated pH and carbonation control systems are designed to continuously monitor the pH of the medium and modulate the supply of CO_2_, with the objective of maintaining optimal conditions for photosynthesis (typically pH 7.5–8.5). Advanced strategies involve the implementation of predictive algorithms that anticipate variations in CO_2_ demand on the basis of diurnal patterns of irradiance, thereby optimizing carbon utilization efficiency [55].

Energy consumption continues to be a significant constraint on commercial viability, particularly in the context of low-value-added applications. The energy consumption of harvesting microalgae biomass by membrane filtration can reach 4.2 kWh kg^−1^ of dry biomass (equivalent to 0.87 kWh m^−3^), with total cultivation costs estimated at 0.30 USD kg^−1^ of dry biomass [74]. While these metrics represent advancements over conventional methods, such as centrifugation, the energy value of the end product imposes stringent limitations on consumption during intermediate processes [74,75].

Systems that recover hydraulic energy from permeate through devices such as turbochargers or pressure exchangers have the potential for a 30–40% reduction in net energy consumption. However, their implementation on a small and medium scale remains challenging [45].

Compared with centrifugation alone, integrated membrane-centrifugation systems have demonstrated a 70% reduction in the total cost of ownership, signifying considerable savings in large-scale operations. This decline can be attributed to a decrease in energy demand, reduced maintenance costs, and enhanced efficiency in the utilization of space and resources [76].

This analysis indicates that while membrane-based systems may incur higher initial capital expenditures, their benefits in terms of productivity, energy efficiency, and resource utilization frequently yield substantial economic advantages over time. Membrane technologies for microalgae are evolving, with advances already enabling energy cost reductions and increased productivity [8,77,78]. The trend is toward greater economic viability as challenges such as fouling and process integration are overcome, making microalgae production more competitive and sustainable [77].

## 6. Environmental and Industrial Implications

The incorporation of membrane technologies into microalgae-based systems has the potential to substantially improve environmental sustainability and encourage industrial innovation. The utilization of customized membranes and hybrid systems facilitates efficient and cost-effective separation, purification, and concentration of biomass and byproducts. This process enhances the production of biofuels, bioproducts, and high-value compounds [8].

Hybrid systems, such as ultrafiltration–osmosis, efficiently remove dissolved organic carbon (up to 99.6%) and other contaminants, outperforming conventional MBRs. They also avoid salinity buildup and produce water streams with different reuse possibilities [79]. MBRs remove more than 90% of oil, grease, and COD, even under saline conditions, making them effective for treating complex wastewater from these sectors [80]. MPBRs with microalgae capture CO_2_ from industrial gas streams, achieving up to 80% removal, while producing valuable biomass. Hybrid systems with microbial fuel cells or photocatalytic structures further increase the rate of carbon sequestration and can generate electricity [31,81].

From an environmental perspective, the integration of microalgal biotechnology with membrane technologies has been demonstrated to directly contribute to the United Nations Sustainable Development Goals (SDGs), particularly SDG 6 (Clean Water and Sanitation), SDG 7 (Affordable and Clean Energy), SDG 9 (Industry, Innovation and Infrastructure), SDG 12 (Responsible Consumption and Production), and SDG 13 (Climate Action). By facilitating more efficient processes with reduced energy consumption and minimal environmental impact, these technologies promote a systemic transition toward a low-carbon circular economy (Table 4).

Consequently, the innovations discussed in this article not only advance the technical frontier of membrane technologies but also provide direction for industrial sectors to meet emerging environmental standards, improve sustainability metrics, and contribute significantly to global climate goals.

## 7. Conclusions and Future Perspectives

Membrane technologies are emerging as transformative tools to overcome critical bottlenecks in microalgae-based systems, particularly in terms of biomass production, carbon capture, and effluent treatment. MPBRs have demonstrated biomass yields that are up to nine times greater than those of conventional systems and promote a 77% reduction in the water footprint and significant gains in energy efficiency. Advanced configurations further increase operational stability, reducing maintenance costs. In carbon capture applications, hollow fiber membranes and hybrid gas–liquid transfer systems have achieved CO_2_ utilization efficiencies of over 85%. These advances highlight the potential of membrane-based microalgae platforms for environmental remediation and resource recovery applications.

However, challenges remain related to membrane fouling, scalability, the durability of materials, and economic viability. Continued advances in materials science, systems integration, and process automation will be essential to overcome these limitations. The technologies reviewed in this article have strong applicability in industrial sectors such as oil and gas, mining, and environmental management. Their adoption can directly contribute to decarbonization efforts, water reuse, and the development of sustainable biorefineries, supporting the transition to a low-carbon circular bioeconomy. Future innovations should prioritize the integration of these systems on an industrial scale to maximize their environmental and economic impacts.

## Figures and Tables

**Table 1:** Integrated comparison of microalgae cultivation systems with membrane technologies.

System	Biomass Concentration (g·L^−1^)	Volumetric Productivity (g·m^−2^·d^−1^)	Energy Consumption (kWh·m^−3^)	Operating Costs	Water Consumption	Reference
MPBR	0.6–1.0	5.08	0.84–0.91	Economically viable	Reduction of up to 77%	[17]
MPBR with MMV	0.7–1.4	-	0.77–0.84	Economically attractive	Similar to MPBR	[19]
Adhered cultivation	Up to 10× higher than suspended system	50–80	-	Reduction in cultivation costs	Minimum water requirement	[20]
AMBR	Up to 5.0	-	-	Reduction in nutrient and treatment costs	Reuse of wastewater	[10,21]

MPBR: Membrane photobioreactors; MMV: magnetically induced membrane vibration; AMBR: algae-based membrane bioreactor.

**Table 2:** Relationships between hydraulic retention time (HRT) and solid retention time (SRT) and the occurrence of membrane fouling, along with the adopted control strategies.

Species	Wastewater Type	HRT (d)	SRT (d)	Membrane Type/Material	Aeration Rate (L min^−1^)	Permeate Flux (L m^−2^ h^−1^)	Fouling Effect	Control Strategy	Ref.
*C. vulgaris*	Synthetic municipal sewage	1 to 5	5 to 25	Flat-sheet, chlorinated PE	0.003	2.6–13	Flux-induced fouling at short HRT	Air scouring for fouling control	[18]
*C. vulgaris* (CPCC 90)	Synthetic municipal wastewater	2.9	50	Flat sheet, PVDF (0.1 µm)	7.5	7.3	** SMP induced fouling mitigated by larger flocs and reduced EPS	Biomass harvesting, flocculation, and SMP control	[58]
*C. vulgaris* (CS-42)	Synthetic municipal wastewater (secondary effluents)	1	18	Hollow fiber, PVDF (0.04 µm)	2.0	4.5	Transmembrane pressure rise due to low filterability and biopolymer-induced biocake	Reduced SRT and culture control to limit biopolymer production	[60]
Indigenous microalgae–bacteria consortia (*Chlorella*, *Scenedesmus*, *Nitzschia*, *Navicula*)	Domestic secondary effluent (activated sludge system)	0.75, 2, and 5	20, 40, and 80	Hollow fiber PVDF (0.04 μm)	6 (5 L/min intermittent + 1 L/min constant)	10	*** TMP rise from protein-rich * BPCs and biocake under high SRT/HRT (40 d/0.75 d) conditions	SRT extension (80 d) and moderate HRT (2–5 d) to reduce BPCs; physical cleaning and aeration to control biocake	[59]
*Spirulina* sp. TISTR 8875	MBR-treated municipal wastewater	6.4 to 9.7	20, 40, 60, 80 d, and infinite (no sludge removal)	Submerged flat-sheet microfiltration membranes (0.4 μm)	4–6	~0.83	Stable operation with controlled fouling	Passive control via high SRT, aeration, and lighting conditions.	[23]

*: Biopolymer clusters; **: soluble microbial products; ***: transmembrane pressure.

**Table 3:** Comparative performance of membrane–microalgae systems.

System	Strain	Substrate or Application	Biomass Production (g/L)	Removal		Energy Demand	Reference
				CO_2_	Nitrogen		
MPBR	*C. vulgaris*	Gas stream (15% CO_2_)	–	80%	–	–	[31]
SFDM–MPBR	*C. vulgaris*	Synthetic wastewater	–	–	100% (nitrate)	–	[31]
HFMPB	*S. platensis*	2% CO_2_ + wastewater	2.1	85%	68%	Low pressure	[62]
MPBR + biofilm	Mixed culture	Biogas (CH_4_/CO_2_)	–	+12% CO_2_ fix.	–	–	[65]

**Table 4:** Membrane technology contributions to the United Nations Sustainable Development Goals (SDGs).

Integrated Application	Related SDGs	Contribution of Membrane—Microalgae Systems	Reference
Bioenergy and Biofuels	7, 13	CO_2_ biofixation and biomass valorization for renewable fuel production	[82,83]
Wastewater Treatment	6, 12, 14	Nutrient removal, water reuse, and effluent polishing Via MPBRs	[8,10]
Food and Feed Applications	2, 12	Biomass for feed/supplements; limited food applications	[83,84,85]
Circular Economy and Resource Recovery	9, 12	CO_2_ capture Integration, nutrient recycling, and waste minimization	[8,83]

## Data Availability

The raw data supporting the conclusions of this article will be made available by the authors on request.

## References

[1] Costa J.A.V., Freitas B.C.B., Moraes L., Zaparoli M., Morais M.G. (2020). Progress in the Physicochemical Treatment of Microalgae Biomass for Value-Added Product Recovery. Bioresour. Technol..

[2] Wang X., Zhang Y., Xia C., Alqahtani A., Sharma A., Pugazhendhi A. (2023). A Review on Optimistic Biorefinery Products: Biofuel and Bioproducts from Algae Biomass. Fuel.

[3] Saha S., Xaxa D.S., Ghosh S.K., Maiti M.K. (2025). Enhanced Accumulation of Important Bioproducts in *Chlorella vulgaris* through AGPase Gene Silencing Coupled with Polyethylene Glycol Treatment. J. Biotechnol..

[4] Ezhumalai G., Arun M., Manavalan A., Rajkumar R., Heese K. (2024). A Holistic Approach to Circular Bioeconomy Through the Sustainable Utilization of Microalgal Biomass for Biofuel and Other Value-Added Products. Microb. Ecol..

[5] Okeke E.S., Ejeromedoghene O., Okoye C.O., Ezeorba T.P.C., Nyaruaba R., Ikechukwu C.K., Oladipo A., Orege J.I. (2022). Microalgae Biorefinery: An Integrated Route for the Sustainable Production of High-Value-Added Products. Energy Convers. Manag. X.

[6] Grobbelaar J.U., Richmond A., Hu Q. (2013). Inorganic Algal Nutrition. Handbook of Microalgal Culture.

[7] Esteves A.F., Pires J.C.M., Gonçalves A.L. (2021). Current Utilization of Microalgae in the Food Industry beyond Direct Human Consumption. Cultured Microalgae for the Food Industry.

[8] Zhao Z., Muylaert K., Vankelecom I.F.J. (2023). Applying Membrane Technology in Microalgae Industry: A Comprehensive Review. Renew. Sustain. Energy Rev..

[9] Gerardo M.L., Aljohani N.H.M., Oatley-Radcliffe D.L., Lovitt R.W. (2015). Moving towards Sustainable Resources: Recovery and Fractionation of Nutrients from Dairy Manure Digestate Using Membranes. Water Res..

[10] François M., Lin K.-S., Rachmadona N. (2025). Microalgae-Based Membrane Bioreactor for Wastewater Treatment, Biogas Production, and Sustainable Energy: A Review. Environ. Res..

[11] Chanquia S.N., Vernet G., Kara S. (2022). Photobioreactors for Cultivation and Synthesis: Specifications, Challenges, and Perspectives. Eng. Life Sci..

[12] Nasser T., Emamshoushtari M.M., Helchi S., Saeidi A., Pajoum Shariati F. (2024). Mitigating Membrane Fouling in an Internal Loop Airlift Membrane Photobioreactor Containing *Spirulina platensis*: Effects of Riser Cross-Sectional Area and Hydrophilic Baffles. Prep. Biochem. Biotechnol..

[13] Bilad M.R., Arafat H.A., Vankelecom I.F.J. (2014). Membrane Technology in Microalgae Cultivation and Harvesting: A Review. Biotechnol. Adv..

[14] Fan L.H., Zhang Y.T., Zhang L., Chen H.L. (2008). Evaluation of a Membrane-Sparged Helical Tubular Photobioreactor for Carbon Dioxide Biofixation by *Chlorella vulgaris*. J. Memb. Sci..

[15] Lai Y.S., Eustance E., Shesh T., Rittmann B.E. (2020). Enhanced Carbon-Transfer and -Utilization Efficiencies Achieved Using Membrane Carbonation with Gas Sources Having a Range of CO_2_ Concentrations. Algal Res..

[16] Kim H.W., Marcus A.K., Shin J.H., Rittmann B.E. (2011). Advanced Control for Photoautotrophic Growth and CO_2_-Utilization Efficiency Using a Membrane Carbonation Photobioreactor (MCPBR). Environ. Sci. Technol..

[17] Bilad M.R., Discart V., Vandamme D., Foubert I., Muylaert K., Vankelecom I.F.J. (2014). Coupled Cultivation and Pre-Harvesting of Microalgae in a Membrane Photobioreactor (MPBR). Bioresour. Technol..

[18] Marbelia L., Bilad M.R., Passaris I., Discart V., Vandamme D., Beuckels A., Muylaert K., Vankelecom I.F.J. (2014). Membrane Photobioreactors for Integrated Microalgae Cultivation and Nutrient Remediation of Membrane Bioreactors Effluent. Bioresour. Technol..

[19] Bilad M.R., Discart V., Vandamme D., Foubert I., Muylaert K., Vankelecom I.F.J. (2013). Harvesting Microalgal Biomass Using a Magnetically Induced Membrane Vibration (MMV) System: Filtration Performance and Energy Consumption. Bioresour. Technol..

[20] Liu T., Wang J., Hu Q., Cheng P., Ji B., Liu J., Chen Y., Zhang W., Chen X., Chen L. (2013). Attached Cultivation Technology of Microalgae for Efficient Biomass Feedstock Production. Bioresour. Technol..

[21] Goh P.S., Ahmad N.A., Lim J.W., Liang Y.Y., Kang H.S., Ismail A.F., Arthanareeswaran G. (2022). Microalgae-Enabled Wastewater Remediation and Nutrient Recovery through Membrane Photobioreactors: Recent Achievements and Future Perspective. Membranes.

[22] González-Camejo J., Aparicio S., Jiménez-Benítez A., Pachés M., Ruano M.V., Borrás L., Barat R., Seco A. (2020). Improving Membrane Photobioreactor Performance by Reducing Light Path: Operating Conditions and Key Performance Indicators. Water Res..

[23] Theepharaksapan S., Lerkmahalikit Y., Namyuang C., Ittisupornrat S. (2023). Performance of Membrane Photobioreactor for Integrated *Spirulina* Strain Cultivation and Nutrient Removal of Membrane Bioreactor Effluent. J. Environ. Chem. Eng..

[24] Novoa A.F., Vrouwenvelder J.S., Fortunato L. (2021). Membrane Fouling in Algal Separation Processes: A Review of Influencing Factors and Mechanisms. Front. Chem. Eng..

[25] Mora-Sánchez J.F., Ribes J., González-Camejo J., Seco A., Ruano M.V. (2024). Towards Optimisation of Microalgae Cultivation through Monitoring and Control in Membrane Photobioreactor Systems. Water.

[26] Yang M., Liu Z., Wang A., Nopens I., Hu H., Chen H. (2024). High Biomass Yields of *Chlorella protinosa* with Efficient Nitrogen Removal from Secondary Effluent in a Membrane Photobioreactor. J. Environ. Sci..

[27] Honda R., Teraoka Y., Noguchi M., Yang S. (2017). Optimization of Hydraulic Retention Time and Biomass Concentration in Microalgae Biomass Production from Treated Sewage with a Membrane Photobioreactor. J. Water Environ. Technol..

[28] Liu S., Rouquié C., Lavenant L., Frappart M., Couallier E. (2022). Coupling Bead-Milling and Microfiltration for the Recovery of Lipids and Proteins from *Parachlorella kessleri*: Impact of the Cell Disruption Conditions on the Separation Performances. Sep. Purif. Technol..

[29] Gkotsis P.K., Zouboulis A.I. (2019). Biomass Characteristics and Their Effect on Membrane Bioreactor Fouling. Molecules.

[30] Penloglou G., Pavlou A., Kiparissides C. (2024). Recent Advancements in Photo-Bioreactors for Microalgae Cultivation: A Brief Overview. Processes.

[31] Senatore V., Buonerba A., Zarra T., Oliva G., Belgiorno V., Boguniewicz-Zablocka J., Naddeo V. (2021). Innovative Membrane Photobioreactor for Sustainable CO_2_ Capture and Utilization. Chemosphere.

[32] Onur A., Ng A., Batchelor W., Garnier G. (2018). Multi-Layer Filters: Adsorption and Filtration Mechanisms for Improved Separation. Front. Chem..

[33] Deng E., Chen X., Rub D., Tran T.N., Lin H. (2022). Energy-Efficient Membranes for Microalgae Dewatering: Fouling Challenges and Mitigation Strategies. Sep. Purif. Technol..

[34] Zarra T., Senatore V., Zorpas A.A., Oliva G., Voukkali I., Belgiorno V., Naddeo V. (2024). Advanced Membrane Photobioreactors in Algal CO_2_ Biofixation and Valuable Biomass Production: Integrative Life Cycle Assessment and Sustainability Analysis. Sustain. Chem. Pharm..

[35] Nguyen T.-T., Bui X.-T., Ngo H.H., Nguyen T.-T.-D., Nguyen K.-Q., Nguyen H.-H., Huynh K.-P.-H., Némery J., Fujioka T., Duong C.H. (2021). Nutrient Recovery and Microalgae Biomass Production from Urine by Membrane Photobioreactor at Low Biomass Retention Times. Sci. Total Environ..

[36] Luo Y., Le-Clech P., Henderson R.K. (2018). Assessment of Membrane Photobioreactor (MPBR) Performance Parameters and Operating Conditions. Water Res..

[37] Rodriguez-Sanchez A., Leyva-Diaz J.C., Gonzalez-Lopez J., Poyatos J.M. (2018). Membrane Bioreactor and Hybrid Moving Bed Biofilm Reactor-Membrane Bioreactor for the Treatment of Variable Salinity Wastewater: Influence of Biomass Concentration and Hydraulic Retention Time. Chem. Eng. J..

[38] Gholizadeh S., Allahyari Z., Carter R.N., Delgadillo L.F., Blaquiere M., Nouguier-Morin F., Marchi N., Gaborski T.R. (2020). Robust and Gradient Thickness Porous Membranes for In Vitro Modeling of Physiological Barriers. Adv. Mater. Technol..

[39] Zhang Y., Ma R., Chu H., Zhou X., Yao T., Zhang Y. (2022). Evaluation of the Performance of Different Membrane Materials for Microalgae Cultivation on Attached Biofilm Reactors. RSC Adv..

[40] Mehariya S., Goswami R.K., Verma P., Lavecchia R., Zuorro A. (2021). Integrated Approach for Wastewater Treatment and Biofuel Production in Microalgae Biorefineries. Energies.

[41] Meng Y., Li A., Li H., Shen Z., Ma T., Liu J., Zhou Z., Feng Q., Sun Y. (2021). Effect of Membrane Blocking on Attached Cultivation of Microalgae. J. Clean. Prod..

[42] Wang R.-L., Li M.-J., Martin G.J.O., Kentish S.E. (2025). Enhancing Direct Air Carbon Capture into Microalgae: A Membrane Sparger Design with Carbonic Anhydrase Integration. Algal Res..

[43] Xu X., Martin G.J.O., Kentish S.E. (2019). Enhanced CO_2_ Bio-Utilization with a Liquid-Liquid Membrane Contactor in a Bench-Scale Microalgae Raceway Pond. J. CO_2_ Util..

[44] Kerner M., Wolff T., Brinkmann T. (2024). Efficient Supply with Carbon Dioxide from Flue Gas during Large Scale Production of Microalgae: A Novel Approach for Bioenergy Facades. Bioresour. Technol..

[45] Zhang M., Yao L., Maleki E., Liao B.-Q., Lin H. (2019). Membrane Technologies for Microalgal Cultivation and Dewatering: Recent Progress and Challenges. Algal Res..

[46] Zheng Q., Martin G.J.O., Kentish S.E. (2019). The Effects of Medium Salinity on the Delivery of Carbon Dioxide to Microalgae from Capture Solvents Using a Polymeric Membrane System. J. Appl. Phycol..

[47] Moraes L., Rosa G.M., Santos L.O., Costa J.A.V. (2020). Innovative Development of Membrane Sparger for Carbon Dioxide Supply in Microalgae Cultures. Biotechnol. Prog..

[48] Pasichnyk M., Stanovsky P., Polezhaev P., Zach B., Šyc M., Bobák M., Jansen J.C., Přibyl M., Bara J.E., Friess K. (2023). Membrane Technology for Challenging Separations: Removal of CO_2_, SO_2_ and NO_x_ from Flue and Waste Gases. Sep. Purif. Technol..

[49] Gkotsis P., Peleka E., Zouboulis A. (2023). Membrane-Based Technologies for Post-Combustion CO_2_ Capture from Flue Gases: Recent Progress in Commonly Employed Membrane Materials. Membranes.

[50] He X. (2021). Polyvinylamine-Based Facilitated Transport Membranes for Post-Combustion CO_2_ Capture: Challenges and Perspectives from Materials to Processes. Engineering.

[51] Han Y., Yang Y., Winston Ho W.S. (2020). Recent Progress in the Engineering of Polymeric Membranes for CO_2_ Capture from Flue Gas. Membranes.

[52] Klinthong W., Yang Y.-H., Huang C.-H., Tan C.-S. (2015). A Review: Microalgae and Their Applications in CO_2_ Capture and Renewable Energy. Aerosol Air Qual. Res..

[53] Costa J.A.V., de Morais M.G., Radmann E.M., Santana F.B., Camerini F., de Souza M.d.R.A.Z., Henrard A.A., da Rosa A.P.C., Brusch L. (2015). Biofixation of Carbon Dioxide from Coal Station Flue Gas Using *Spirulina* sp. LEB 18 and *Scenedesmus obliquus* LEB 22. African J. Microbiol. Res..

[54] Duarte J.H., Fanka L.S., Costa J.A.V. (2016). Utilization of Simulated Flue Gas Containing CO_2_, SO_2_, NO and Ash for *Chlorella fusca* Cultivation. Bioresour. Technol..

[55] Hou Y., Han T., Wu R., Liu Z., Ma Y., Guo Z., Hao N., Wang W., Ji X., Zhu Z. (2023). A Novel System Integrating Electrolysis and Ionic Membranes (EIMs) Enables Artificial Carbon Concentration and Alleviation of Metal Cation Stress in Microalgae Cultivation. Green Chem..

[56] Kim G.Y., Heo J., Kim K., Chung J., Han J.I. (2021). Electrochemical pH Control and Carbon Supply for Microalgae Cultivation. Chem. Eng. J..

[57] Luo Y., Le-Clech P., Henderson R.K. (2017). Simultaneous Microalgae Cultivation and Wastewater Treatment in Submerged Membrane Photobioreactors: A Review. Algal Res..

[58] Zou H., Rutta N.C., Chen S., Zhang M., Lin H., Liao B. (2022). Membrane Photobioreactor Applied for Municipal Wastewater Treatment at a High Solids Retention Time: Effects of Microalgae Decay on Treatment Performance and Biomass Properties. Membranes.

[59] Segredo-Morales E., González E., González-Martín C., Vera L. (2022). Secondary Wastewater Effluent Treatment by Microalgal-Bacterial Membrane Photobioreactor at Long Solid Retention Times. J. Water Process Eng..

[60] Luo Y., Le-Clech P., Henderson R.K. (2020). Assessing the Performance of Membrane Photobioreactors (MPBR) for Polishing Effluents Containing Different Types of Nitrogen. Algal Res..

[61] Soomro R.R., Ndikubwimana T., Zeng X., Lu Y., Lin L., Danquah M.K. (2016). Development of a Two-Stage Microalgae Dewatering Process—A Life Cycle Assessment Approach. Front. Plant Sci..

[62] Kumar A., Yuan X., Sahu A.K., Dewulf J., Ergas S.J., Van Langenhove H. (2010). A Hollow Fiber Membrane Photo-Bioreactor for CO_2_ Sequestration from Combustion Gas Coupled with Wastewater Treatment: A Process Engineering Approach. J. Chem. Technol. Biotechnol..

[63] Borges J.A., Rosa G.M., Meza L.H.R., Henrard A.A., Souza M.R.A.Z., Costa J.A.V. (2013). *Spirulina* sp. LEB-18 Culture Using Effluent from the Anaerobic Digestion. Braz. J. Chem. Eng..

[64] Takabe Y., Himeno S., Okayasu Y., Minamiyama M., Komatsu T., Nanjo K., Yamasaki Y., Uematsu R. (2017). Feasibility of Microalgae Cultivation System Using Membrane-Separated CO_2_ Derived from Biogas in Wastewater Treatment Plants. Biomass and Bioenergy.

[65] Zhang B., Liu J., Cai C., Zhou Y. (2025). Membrane Photobioreactor for Biogas Capture and Conversion—Enhanced Microbial Interaction in Biofilm. Bioresour. Technol..

[66] Wang R.-L., Li M.-J., Li D., Yang Y.-W. (2022). The Synergy of Light/Fluid Flow and Membrane Modification of a Novel Membrane Microalgal Photobioreactor for Direct Air Carbon Capture. Appl. Energy.

[67] Zhao Z., Muylaert K., Vankelecom I.F.J. (2021). Combining Patterned Membrane Filtration and Flocculation for Economical Microalgae Harvesting. Water Res..

[68] Razak N.N.A.N., Rahmawati R., Bilad M.R., Pratiwi A.E., Elma M., Nawi N.I.M., Jaafar J., Lam M.K. (2020). Finned Spacer for Enhancing the Impact of Air Bubbles for Membrane Fouling Control in *Chlorella vulgaris* Filtration. Bioresour. Technol. Rep..

[69] Zheng M., Yang Y., Qiao S., Zhou J., Quan X. (2021). A Porous Carbon-Based Electro-Fenton Hollow Fiber Membrane with Good Antifouling Property for Microalgae Harvesting. J. Memb. Sci..

[70] Mkpuma V.O., Moheimani N.R., Ennaceri H. (2022). Microalgal Dewatering with Focus on Filtration and Antifouling Strategies: A Review. Algal Res..

[71] Castro-Muñoz R., García-Depraect O. (2021). Membrane-Based Harvesting Processes for Microalgae and Their Valuable-Related Molecules: A Review. Membranes.

[72] Liao Y., Bokhary A., Maleki E., Liao B. (2018). A Review of Membrane Fouling and Its Control in Algal-Related Membrane Processes. Bioresour. Technol..

[73] Marchese A., Lima S., Cosenza A., Giambalvo F., Scargiali F. (2025). Effects of Light Quality Adjustment in Microalgal Cultivation: Flashing Light and Wavelength Shifts in Photobioreactor Design. Processes.

[74] Wang L., Pan B., Gao Y., Li C., Ye J., Yang L., Chen Y., Hu Q., Zhang X. (2019). Efficient Membrane Microalgal Harvesting: Pilot-Scale Performance and Techno-Economic Analysis. J. Clean. Prod..

[75] Monte J., Sá M., Galinha C.F., Costa L., Hoekstra H., Brazinha C., Crespo J.G. (2018). Harvesting of *Dunaliella salina* by Membrane Filtration at Pilot Scale. Sep. Purif. Technol..

[76] Monte J., Bernardo J., Sá M., Parreira C., Galinha C.F., Costa L., Casanovas C., Brazinha C., Crespo J.G. (2020). Development of an Integrated Process of Membrane Filtration for Harvesting Carotenoid-Rich *Dunaliella salina* at Laboratory and Pilot Scales. Sep. Purif. Technol..

[77] Ennaceri H., Fischer K., Schulze A., Moheimani N.R. (2022). Membrane Fouling Control for Sustainable Microalgal Biodiesel Production: A Review. Renew. Sustain. Energy Rev..

[78] Zhao Z., Blockx J., Muylaert K., Thielemans W., Szymczyk A., Vankelecom I.F.J. (2022). Exploiting Flocculation and Membrane Filtration Synergies for Highly Energy-Efficient, High-Yield Microalgae Harvesting. Sep. Purif. Technol..

[79] Moser P.B., dos Anjos Silva G.R., Lima L.S.F., Moreira V.R., Lebron Y.A.R., de Paula E.C., Amaral M.C.S. (2021). Effect of Organic and Inorganic Draw Solution on Recalcitrant Compounds Build up in a Hybrid Ultrafiltration-Osmotic Membrane Reactor Treating Refinery Effluent. Chem. Eng. J..

[80] Fulazzaky M., Setiadi T., Fulazzaky M.A. (2020). An Evaluation of the Oilfield-Produced Water Treatment by the Membrane Bioreactor. J. Environ. Chem. Eng..

[81] Huang D., Li M.-J., Wang R.-L., Yang Y.-W., Tao W.-Q. (2021). Advanced Carbon Sequestration by the Hybrid System of Photobioreactor and Microbial Fuel Cell with Novel Photocatalytic Porous Framework. Bioresour. Technol..

[82] Merlo S., Gabarrell Durany X., Pedroso Tonon A., Rossi S. (2021). Marine Microalgae Contribution to Sustainable Development. Water.

[83] Sarker N.K., Kaparaju P. (2024). Microalgal Bioeconomy: A Green Economy Approach Towards Achieving Sustainable Development Goals. Sustainability.

[84] Sutherland D.L., McCauley J., Labeeuw L., Ray P., Kuzhiumparambil U., Hall C., Doblin M., Nguyen L.N., Ralph P.J. (2021). How Microalgal Biotechnology Can Assist with the UN Sustainable Development Goals for Natural Resource Management. Curr. Res. Environ. Sustain..

[85] Agarwalla A., Mohanty K. (2024). A Critical Review on the Application of Membrane Technology in Microalgal Harvesting and Extraction of Value-Added Products. Sep. Purif. Technol..

